# Global VAX: A U.S. contribution to global COVID-19 vaccination efforts, 2021–2023

**DOI:** 10.1016/j.vaccine.2024.03.054

**Published:** 2024-03-23

**Authors:** Benjamin A. Dahl, Beth Tritter, Deena Butryn, Melissa Dahlke, Sean Browning, Richard Gelting, Monica Fleming, Nancy Ortiz, Jacqueline Labrador, Ryan Novak, David Fitter, Elizabeth Bell, Megan McGuire, Robert Rosenbaum, Robert Pulwer, Jolene Wun, Anna McCaffrey, Maisoon Chowdhury, Nida Parks, Marc Cunningham, Anthony Mounts, Dora Curry, Dominique Richardson, Gavin Grant

**Affiliations:** a Centers for Disease Control and Prevention, United States; b US Agency for International Development, United States; c Taskforce for Global Health, United States

**Keywords:** COVID-19, Vaccine, SARS-CoV-2, Coronavirus disease, Evaluation, Implementation, Vaccine delivery, Global Vax

## Abstract

In December 2021 the U.S. Government announced a new, whole-of-government $1.8 billion effort, the Initiative for Global Vaccine Access (Global VAX) in response to the global COVID-19 pandemic. Using the foundation of decades of U.S. government investments in global health and working in close partnership with local governments and key global and multilateral organizations, Global VAX enabled the rapid acceleration of the global COVID-19 vaccine rollout in selected countries, contributing to increased COVID-19 vaccine coverage in some of the world’s most vulnerable communities. Through Global VAX, the U.S. Government has supported 125 countries to scale up COVID-19 vaccine delivery and administration while strengthening primary health care systems to respond to future health crises. The progress made by Global VAX has paved the way for a stronger global recovery and improved global health security.

## 1. Introduction

**Fig. 1.**
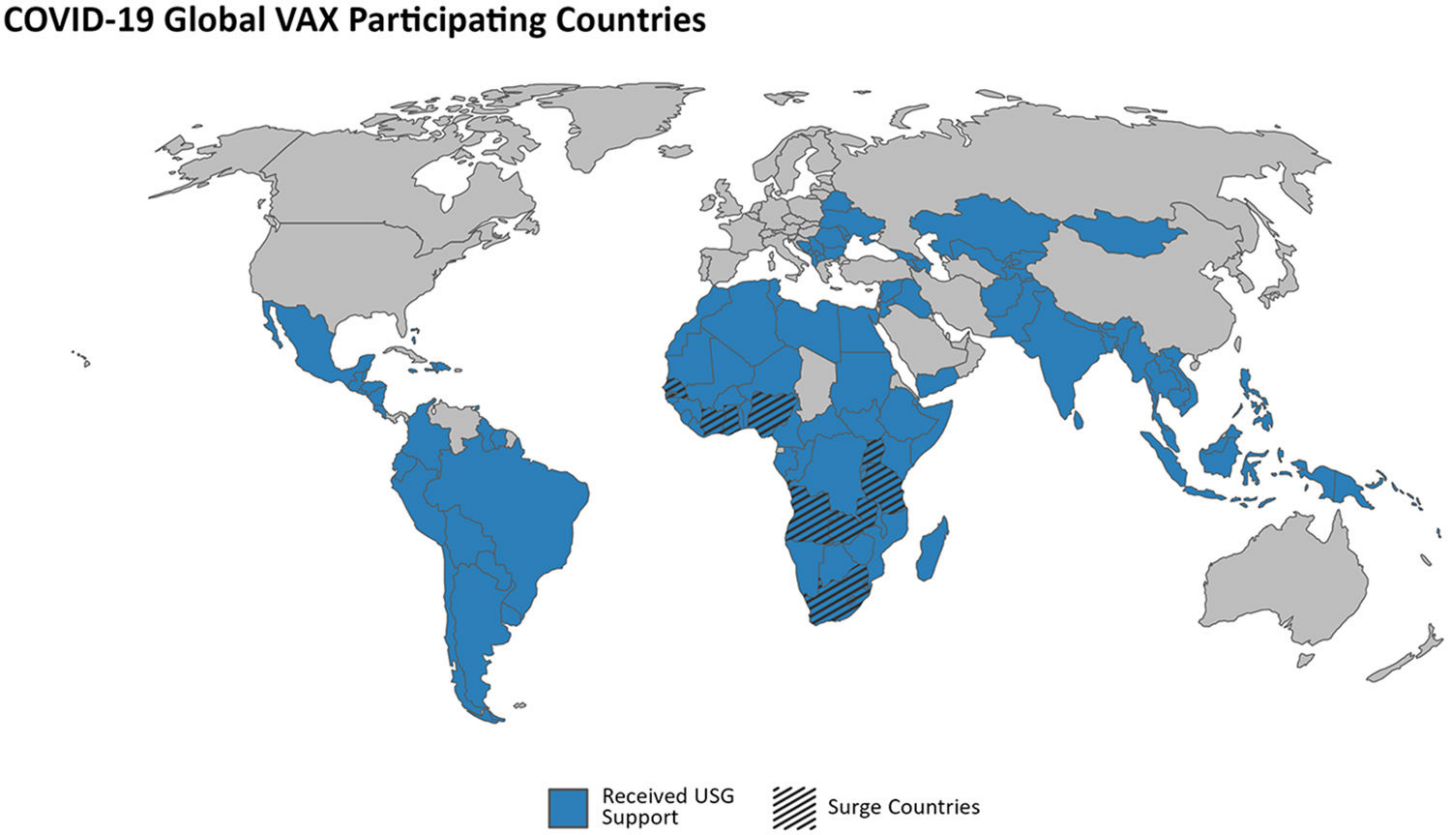
Map.

The first COVID-19 vaccines were introduced in December 2020, but global availability was limited and that was concentrated in high- and upper-middle-income countries. By the end of 2021, the global community had manufactured and made available sufficient supply of COVID-19 vaccines, but significant challenges remained for country programs to administer the vaccines to priority populations. To address this major gap and complement its investments in global supply of COVID-19 vaccines, the U.S. Government (USG) launched a $1.8 billion Initiative for Global Vaccine Access (Global VAX) [1,2] in December 2021 to support countries to rapidly accelerate COVID-19 vaccine delivery and uptake. Global VAX mobilized the capabilities of multiple USG entities including United States Agency for International Development (USAID); Centers for Disease Control and Prevention (CDC); Department of State, including the Office of the Global AIDS Coordinator (S/GAC); Department of Defense (DoD); Department of Health and Human Services (HHS); Peace Corps; the U.S. International Development Finance Corporation; Department of Treasury; and other interagency partners under a landmark whole-of-government effort to support the delivery and administration of COVID-19 vaccines in 125 countries [1] (Fig. 1). Global VAX built upon existing partnerships with key multilateral partners—including the World Health Organization (WHO); Gavi, the Vaccine Alliance; the COVID-19 Vaccines Global Access (COVAX) facility; UNICEF; and others—to harmonize COVID-19 vaccine donations, deliveries, and plans for vaccine rollout and administration.

The main priority of Global VAX has been to provide financial, technical, and diplomatic support to countries to accelerate uptake of COVID-19 vaccines and help achieve vaccination coverage targets, thereby providing greater population level protection from severe COVID-19 disease. Among the 125 countries that received Global Vax support, a total of 11 high priority ‘surge’ countries were identified to receive intensive support: Angola, Côte d’Ivoire, Eswatini, Ghana, Lesotho, Nigeria, Senegal, South Africa, Tanzania, Uganda, and Zambia. The surge countries were selected based on a variety of quantitative and qualitative factors, including the burden of COVID-19 on a country’s population; a country’s ability to administer and deliver vaccines in the absence of supply constraints; the current capacity of a country’s health system; the opportunity for additional U.S. investments to be used effectively and rapidly; and the strength of our longstanding partnership.

### 1.1. Principles and practices

Since December 2021, Global VAX has been the coordinated USG effort to accelerate COVID-19 vaccine uptake aligned with World Health Organization (WHO) and member country targets and prioritization strategies. Global VAX has supported a range of activities to increase equitable vaccination among priority populations, including those living in hard-to-reach places, and to increase demand for vaccinations. Global VAX has been guided by the following principles:

- Prioritize the most effective and efficient approaches to COVID-19 vaccine delivery
- Leverage existing global health platforms and partners when possible
- Empower those working in the countries to develop country- and region-specific approaches to COVID-19 vaccine delivery

To maximize impact, USG agencies have worked together at country and headquarter levels to coordinate and implement vaccination efforts. At the headquarters level, USAID and CDC [3] have led day-to-day operational coordination: facilitating collaborative country planning and interagency coordination; hosting a shared dashboard for tracking progress and key metrics; and coordinating policy engagement, collaboration, and communication with external stakeholders. The USG also convened a Global VAX Interagency Council, composed of decisionmakers from all relevant USG agencies, for regular planning meetings to address urgent matters from the field related to implementation. Council members have been responsible for providing policy-level guidance on the initiatives’ goals, objectives, and implementation. Agencies have collaborated on Global VAX program planning, with each agency maintaining final authority over and accountability for its own funding.

Specific technical support was coordinated with Governments at the country level. USAID, CDC, State, and other relevant agencies have directly planned for the programming of their own appropriated resources in mutual coordination with each other, building upon previous COVID-19 and other global health investments. Examples of this coordinated programming have included investing in cold chain and supply logistics to safely store and deliver vaccines; supporting country-specific vaccination campaigns; launching mobile vaccination sites for hard-to-reach, priority, and rural populations; assisting in vaccine policymaking and planning; and supporting development of health information systems to evaluate vaccine equity and safety. USG country teams, working with ministries of health and local partners, developed Global VAX Acceleration Plans inclusive of all USG funding and programming that aligned with and contributed to partner countries’ national priorities. USAID, in collaboration with CDC, and in alignment with WHO guidelines [4], also developed and disseminated technical guidance to field teams for the development and implementation of these plans. The accelerated vaccine uptake plans have included vaccination campaigns as well as expanding the immunization systems required to support implementation.

Recognizing the need for greater coordination among global partners around COVID-19 vaccine delivery, in early 2022, WHO, UNICEF, and Gavi launched the COVID-19 Vaccine Delivery Partnership (CoVDP) with support from the U.S. government. CoVDP brought together a broad network of global partners, including the Africa Centres for Disease Control and Prevention, the World Bank, the International Monetary Fund, United Nations organizations, the European Union, the G20, and many others. Through USAID and U.S. embassies, the U.S. government provided significant support to CoVDP for global coordination and country readiness and COVID-19 vaccine delivery. CoVDP worked to enhance coordination, reduce transaction costs, and leverage the combined expertise and resources of national and international partners through a “One Team, One Plan, One Budget” approach in each country in which it was active. One Team, led by the host government, was at the center of the approach, using a joint operational plan (One Plan) and a joint assessment of funding needs and availability (One Budget). Coordinating with CoVDP and the One Team in each country allowed Global VAX to align resources and activities in support of country goals and reduce duplication of effort.

As part of the Global VAX initiative, the USG has contributed to the development of technical tools, guidance and policy for COVID-19 vaccination and has supported ministries of health and their partners in developing or strengthening national and sub-national capacities for the planning, implementation, and evaluation of COVID-19 vaccination programs. Technical priority areas and definitions are shown in the box below:

**Table:** 

**Community Engagement, Advocacy, and Demand Generation**	Design and carry out population-specific activities that encourage demand for COVID-19 vaccination and address barriers to uptake of COVID-19 vaccines, including engaging community leaders and decisionmakers to promote understanding and support for equitable distribution of COVID-19 vaccines.
**Supply Chain and Logistics**	Ensure COVID-19 vaccine-specific supply planning, storage, and distribution requirements are blended into existing supply chains and COVID-19 vaccine stock movements are documented.
**Policy, Planning, and Coordination**	Develop, disseminate, and utilize plans, policies, and operational guidelines for COVID-19 vaccination.
**Pharmacovigilance**	Develop and apply guidelines, processes, and tools for detecting, reporting, and investigating adverse events following immunization (AEFI).
**Vaccine Service Delivery**	Identify, design, and expand ways to support delivery of COVID-19 vaccines in urban and rural areas, including through the use of health centers, mass campaigns, mobile sites, and other delivery platforms that reach priority populations.
**Human Resources for Health**	Identify and train staff to carry out COVID-19 vaccination efforts.
**Monitoring, Evaluation, and Health Information Systems**	Design and develop data collection tools and processes to inform decision making around vaccine delivery and to ensure effective oversight of COVID-19 vaccination activities.

The Global VAX reporting process has enabled the careful tracking and monitoring of USG support and has consolidated interagency, implementing partners, and partner government reporting on a set of indicators. Indicator reports have included data from implementing partners such as number of trained community leaders, educators and health workers, number of supported vaccine sites and campaigns, and number of people reached through vaccine-focused messages; and data from global partnerships and publicly accessible data such as vaccine supply, coverage, and implementation (including by high-risk group, and geography), and policy or leadership changes.

These reports have been included in the Global VAX dashboard and reviewed, updated, and shared on a weekly basis to enable all interagency partners to track progress and key metrics. During the initiative, the 11 Global VAX countries receiving surge support have submitted additional interagency progress reports of completed activities, challenges to accelerating vaccination programs, and progress towards targets on a quarterly basis. This quarterly reporting has enabled opportunities to address issues identified, modify activities as needed, and share lessons learned with other countries.

## 2. Outcomes

Through June 2023, the USG has supported the purchase and delivery of over 687 million doses globally of COVID-19 vaccines since they became available in December 2020. Global VAX has complemented this USG effort with a surge of additional technical, financial, and diplomatic support to countries.

**Fig. 2.**
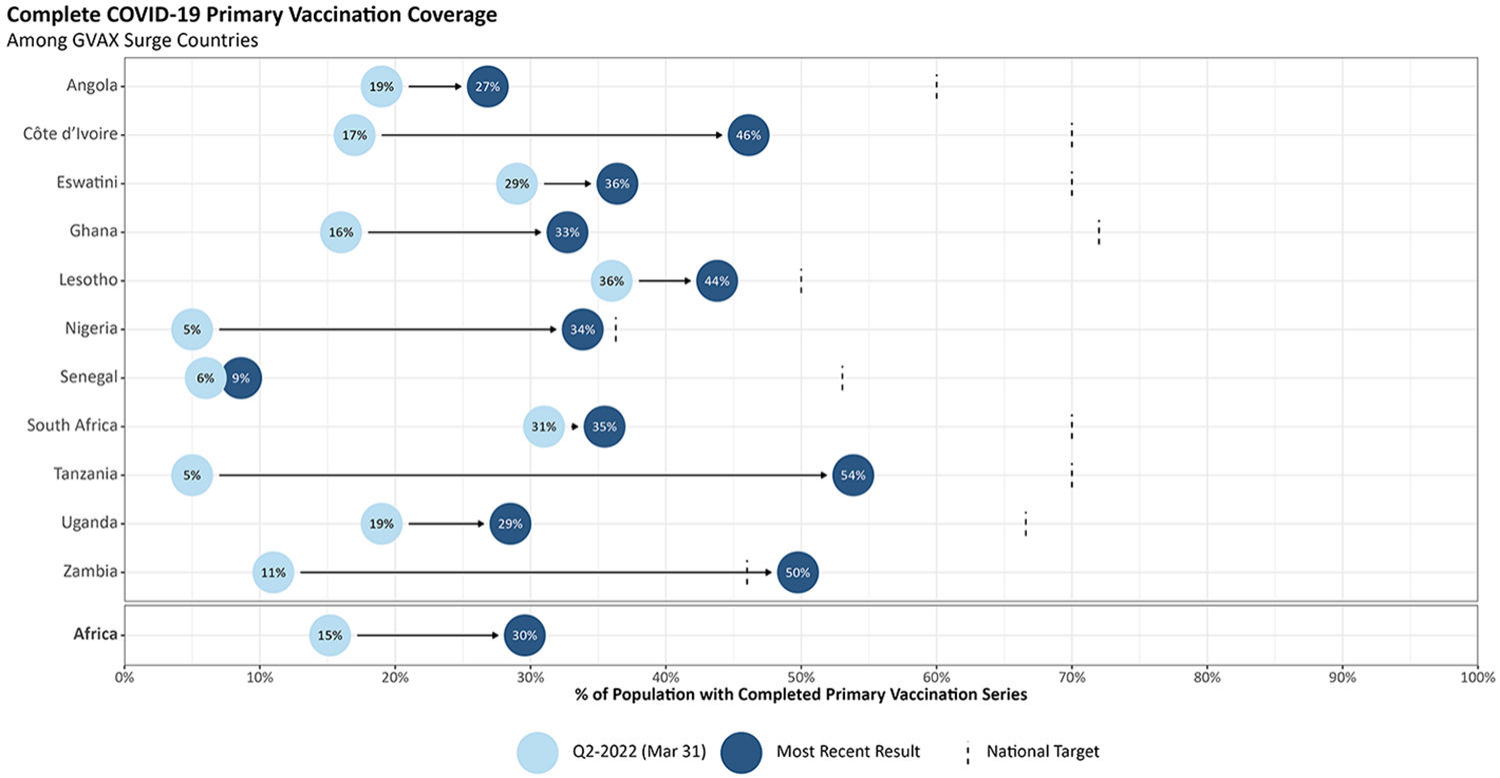
Coverage increase in surge countries and African region overall. Source: COVID-19 Vaccine Delivery Partnership Hub, as of Jun 22, 2023 Our World in Data Coronavirus (COVID-19) Vaccinations, as of Jun 22, 2023

When Global VAX launched in December 2021, the eleven priority countries had reported just 5 % complete primary COVID-19 vaccination coverage of their populations (Fig. 2). As of June 22, 2023, that number has risen to 35 % of their total population—or nearly 60 % of those countries’ eligible populations. In the African region, for example, COVID-19 vaccination coverage increased from 15 % to 30 % during this period. In some countries, though not all, the increase has been even more dramatic. In Tanzania, complete primary coverage rose from just 1.6 % on December 1, 2021 to 54 % on June 22, 2023. Significant local leadership in Tanzania, along with sustained USG interagency diplomatic engagement and substantial USG financial and technical support, played a critical role in providing the ongoing support to rapidly accelerate and sustain COVID-19 vaccinations through Global VAX. By leveraging existing platforms in-country, such as the President’s Emergency Plan for AIDS Relief (PEPFAR), providing an array of technical assistance (see definition box), and mobilizing existing relationships from the grassroots to the highest levels of government, Global VAX has been able to help countries rapidly achieve their vaccination goals.

## 3. Discussion

### 3.1. Successes

- **Focused financial, technical, and diplomatic support for COVID-19 vaccine delivery and administration in 11 countries.** Many of the 11 Global VAX surge countries experienced significant gains in COVID-19 vaccination coverage since the launch of the initiative and are now pivoting to full integration of COVID-19 vaccination across the health system – demonstrating both short-term success during the acute phase of the pandemic and progress in sustaining these investments for the benefit of the primary health care system. The concentration of significant USG resources in these countries allowed Global VAX to coordinate and focus efforts, identify, and disseminate best practices to other countries, and demonstrate the viability of Global VAX programs.
- **Extensive support to the >110 additional countries.** Having a comprehensive set of resources to support vaccine demand, strategic information, logistics. etc., enabled USG to support countries to target critical gaps to enable vaccines to be administered to priority populations.
- **Building on existing in-country agency structures and programs.** The interagency teams leveraged programs that already existed and had significant community support such as US Embassies, PEPFAR, USAID Missions, CDC country offices [6], international Influenza networks [7], Peace Corps and DoD, among others. This allowed for a much more rapid launch of this massive response.
- **Standing up dedicated interagency teams at respective headquarters to support all aspects of the COVID-19 vaccine rollout.** Interagency coordination at agency headquarters provided coordinated support to the country teams, allowing agencies to ensure that country technical, coordination and programmatic needs were met in a changing environment.
- **Tracking indicators spanning the entire COVID-19 vaccine delivery pathway was critical to ensuring that COVID-19 vaccines reach the communities that need them most when they need them most.** When Global VAX launched, many countries did not have sufficient systems in place to track growing national COVID-19 vaccination programs and therefore faced challenges in collecting and analyzing COVID-19 vaccination data. Global VAX supported partner governments in strengthening their monitoring and evaluation systems and used the data generated by these systems to inform planning and decision making, prioritize vulnerable populations, and allow for real-time adjustments.

### 3.2. Challenges

- **National vaccination coverage targets in conflict with eligibility.** Many countries set the goal of 70 % national coverage based on WHO recommendations [4], yet those eligible to receive COVID-19 vaccines had to be ages 18 or older. In countries with large populations under 18 years, meeting coverage targets was not possible.
- **Prevalent vaccine-related rumors and misinformation.** Misinformation and underlying vaccine hesitancy has challenged COVID-19 vaccine roll-out [5] before and after the launch of Global VAX. Demand generation, especially community engagement, remains a key technical need in myriad countries, particularly as the pandemic recedes and as-yet unvaccinated populations remain hesitant. This hesitancy may have long-term consequences on vaccination programs beyond COVID-19.
- **Waning political will for COVID-19 vaccinations in some countries.** As reported COVID-19 case numbers declined, pandemicrelated travel restrictions were removed, and other health and political issues persisted, many foreign government leaders at national and subnational levels de-prioritized the COVID-19 response, including vaccinations. While COVID-19 vaccination coverage continues to increase slightly, gaining support for ongoing COVID-19 vaccination efforts is more difficult, even with ample COVID-19 vaccine supply and large proportions of eligible populations still unvaccinated.
- **Challenges reaching priority populations,** such as the elderly and immunocompromised—who are not served through routine immunization platforms.
- **Impact of pandemic on routine immunizations.** The percentage of children who received three doses of the vaccine against diphtheria, tetanus and pertussis (DTP3) – a common marker for immunization coverage – fell 5 percentage points between 2019 and 2021 to 81 per cent. In absolute numbers, 25 million children missed one or more doses of DTP in 2021 which is 6 million more than in 2019 [8]. This has rebounded some in 2022 (latest data available) but are still behind pre-pandemic levels.

## 4. Conclusion

Since December 2021, the USG has supported 125 countries globally to receive and administer COVID-19 vaccines. Through the Global VAX initiative, the USG efforts have contributed to strengthening countries’ capacity to plan for and deliver COVID-19 vaccines while building longterm, sustainable capacity for immunization programs and response to infectious disease threats. As the world emerges from the emergency response phase of the pandemic, ongoing Global VAX country support is increasingly focused on integrating COVID-19 vaccination efforts into national and sub-national health systems, in line with country priorities. Integrating COVID-19 vaccinations and care into existing platforms helps to safeguard essential health services while boosting health system resiliency to respond to future health emergencies. Building on the lessons learned about the role of vaccination in pandemics, the Global VAX initiative has helped strengthen immunization programs to be increasingly robust to deploy vaccines in emergency situations and to ensure more equitable vaccine access.

## Data Availability

No data was used for the research described in the article.
